# The principle of stewardship truthful communication for the restoration of trust in public health officials

**DOI:** 10.1093/pubmed/fdaf073

**Published:** 2025-11-18

**Authors:** Thana C de Campos-Rudinsky

**Affiliations:** Escuela de Gobierno & Instituto de Eticas Aplicadas, Pontifical Catholic University of Chile, Chile

**Keywords:** accessibility, accountability, communication, public health officials, stewardship, transparency, trust, truthfulness

## Abstract

In the wake of widespread public skepticism toward health authorities, this paper introduces a stewardship model of communication to guide public health officials in restoring trust. In critically examining the broader ethical concept of stewardship as developed by the World Health Organization and the Nuffield Council on Bioethics, the paper argues that public health officials—as stewards of the population’s health and well-being—have an ethical duty to convey scientific knowledge truthfully. Rather than promoting excessive transparency or manipulating public perception through noble lies, the proposed stewardship model insists on honesty tempered by discretion. This entails conveying information that is accessible, assessable, and accountable. Through conceptual analysis and practical examples, the paper outlines how public health officials can fulfill their ethical duty of care by reclaiming their role as trustworthy stewards.

## Introduction

What do good and courageous public health practice and policy mean in a world in turmoil? There is a persistent public distrust in health authorities.^1–3^ This distrust has intensified amidst several public health emergencies, when authorities have not been able to communicate effectively the risks involved and the justifications grounding their interventions.^1^ Restoring public trust in health authorities requires revising the ethics of expert communication,^1^^,^^4^ I argue that public health officials—as stewards of the population’s well-being—have an ethical duty to convey scientific knowledge truthfully: that is, with honesty tempered by discretion, framing messages that are accessible, assessable, and accountable. My stewardship model of good communication advances a tripartite framework for restorative communication that (i) filters technical detail to match audience needs; (ii) discloses uncertainties, trade-offs, and limitations responsibly; and (ii) invites two-way dialogue and shared decision-making. Drawing on concrete examples, the paper offers practical guidelines for embedding stewardship in everyday public health practice. By operationalizing stewardship as an ethic of presence, vulnerability, and empathy, public health officials can rebuild trust and foster genuine community engagement.

## Evolution of the stewardship concept

The World Health Organization (WHO) first articulated the idea of stewardship in its 2000 *World Health Report*, defining it as “the careful and responsible management of the wellbeing of the population.”^5^ In emphasizing the managerial function of stewardship, the WHO situates it alongside good governance, regulatory oversight, and accountability in health-system management.^5^^,^^6^ In this management framing, states and especially their public health officials should act as “effective trustees of national health”^7^ entrusted to make decisions on behalf of citizens and residents in a trustworthy manner.^8^

Building on WHO’s managerial posture, the Nuffield Council on Bioethics subsequently strengthens the theoretical foundations of stewardship by grounding it in John Stuart Mill’s liberal “no-harm” principle.^9^ This liberal approach justifies interventions only when individual actions risk harming others, recommending policies that minimize coercion.^9^ Yet, it also allows for certain policies that promote procedural justice and community well-being, such as safe exercise spaces and addiction support programs.^9^^,^^10^ Crucially, the Council aims to differentiate stewardship from paternalism—the “interference of the state or an individual with another person, against their will”^9^ justified by beneficent aims. Unlike paternalism, stewardship avoids highly coercive universal measures, prioritizing instead “respect for individuality by seeking the least intrusive means to achieve policy goals.”^9^ However, in also aiming to intervene to address some health inequalities effectively, the Council endorses in actuality what they call a "qualified paternalism."^10^ In providing justifications for certain forms of paternalistic interventions,^9^ the Council’s definition of stewardship spurred a protracted scholarly debate.^10–12^

By focusing narrowly on system management, it is indeed unclear how the technocratic conception of stewardship could truly engage individuals’ agency without devolving into paternalistic overreach or “nanny-statism.”^13–15^ This justifies a shift from system-centered management to a people-centered, relational account of stewardship. This paper answers this call for relationality by reframing stewardship as an ethic of expert communication—one that pairs scientific honesty with prudential discretion and mutual engagement. The steward, within this relational framing, is a *servant* entrusted not only with the management of systems but also—and primarily—with the care of the population, their health, and wellbeing. One important way in which the steward cares well for those entrusted to him is by communicating well with them. Hence, the centrality of an effective communication between steward and the ones under his care.

### The stewardship model of good communication: relationality as grounds for good communication

To reimagine stewardship as an ethic of dialog, and not merely as a mandate to manage systems efficiently, I ground my model in three essential ingredients: relational presence, reasonable vulnerability, and empathetic listening. The purpose of the model is good communication—messages conveyed in an accessible, assessable, and accountable manner.

### Accessibility through relational presence

When I offer my full attention to the other before me, I offer him my presence. In being present, I am able to learn about the other with whom I wish to communicate: the language he speaks, his background, his existing knowledge, his capacity for understanding, his concerns, his needs. This attentive presence enables me to make an informed judgement about how best to tailor my message to that specific addressee. Presence therefore is the necessary condition for accessibility: it is what renders a message intelligible, relevant, and engaging to its intended audience. This is the steward’s first and more foundational task.

In practice, this requires public health officials to filter data prudently, to avoid both excessive technical detail (data deluge overwhelms!) and overly abstract generalities (which lose substance).^16^ Filtering must always be tailored to the audience.^17^ A lay audience and a scientific audience require different modes of framing. Yet both need only those data and explanations essential to decision-making; outdated, peripheral, or low-quality material should be omitted.^18^

When communicating with the public, it is key that officials convey (i) that uncertainty is intrinsic to science, and (ii) that scientific progress occurs through disagreement, critique, and debate.^19^ Since the “pluralistic, revisable, and fallible,”^20^ nature of science is not always evident to non-scientists, a more explicit disclosure of uncertainty is typically helpful—for instance, by explaining that even among equally qualified experts, differing interpretations of uncertainties may lead to different conclusions and, therefore, divergent messages.^19^ Such disclosure need not cause confusion or rejection of the proposed interventions, provided the official’s message translates into concrete implications of “What this means for you and your community”, as I will further explore below.

### Assessability through reasonable vulnerability

If attentive presence ensures that the truth is not lost in translation but received as an accessible gift, reasonable vulnerability is what makes that truth assessable—that is, open to examination and verification.^21^ For a message to be tested, evaluated, or trusted, it must be delivered with candor^22^: disclosing uncertainties, trade-offs, causation, and correlation without overwhelming or paralyzing their audience. Honesty therefore must be tempered with discretion: this is where truthfulness happens. Public health officials best serve their communicative role when they speak the truth with both courage and humility—a reasonable degree of vulnerability that strengthens, rather than diminishes, their authority, reinforcing their trustworthiness.

Take, for example, the WHO’s 2021 statement encouraging breastfeeding. It claimed that “breastfeeding improves IQ, school attendance, and is associated with higher income in adult life”.^23^ Though well-intentioned, the statement presented correlation as causation, oversimplifying complex findings and downplaying key confounding factors such as parental education and income.^1^ A stewardship-based message might instead read as follows: “Studies show a correlation between breastfeeding and later academic outcomes; however, many factors—including parental education—also contribute.”

This alternative fosters assessability. It opens a space for shared reasoning, acknowledging the limits of the study (“while the data are adjusted for X and Y, causality cannot be definitively established”). The difference is subtle yet decisive: the first message (the 2021 statement issued by the WHO) intends passive acceptance; the second invites informed engagement and democratic scrutiny. And where engagement and scrutiny are possible, trust can take root.

### Accountability through empathetic listening

If relational presence makes communication accessible, and reasonable vulnerability makes it assessable, then empathetic listening is what renders it accountable. Accountability is not simply a matter of disclosure, nor can it be fulfilled by a box-ticking transparency (in fact, perfunctory accountability leads to excessive disclosure and transparency!).^16^^,^^21^ Instead, effective accountability requires a posture of receptivity: a readiness to respond to those affected by one’s message. This is not just quickly reacting with a defensive reply, but making space for critique, dissent, and moral challenge. When accountability is understood relationally as answerability and responsiveness, communication is not seen as a one-way transmission but a two-way exchange.^24^^,^^25^ The steward does not speak from the protected space of untouchable authority. Rather, she speaks and listens in return, considering public inputs, and recognizing the lived experiences and values of those who she shepherds as valid expertise alongside her technical knowledge. By so doing, the steward honors the shared authority of the relationship.

In practice, empathetic listening^26^ requires public health officials to treat communication not as a final word, but as the beginning of a dialog, where they remain answerable to the people they serve. This may involve adjusting messages that have landed poorly, clarifying when confusion arises, acknowledging the moral weight of public frustration in times of crisis, or apologizing for mistakes. During the COVID-19 pandemic, the absence of empathetic listening was evident. For example, early messages discouraging public mask use (in spite of emerging scientific evidence supporting it) failed to acknowledge the non-scientific constraints shaping policy, namely: mask shortages and the need to prioritize frontline workers.^1^^,^^27^ When the message changed, the public’s utter confusion and suspicion was palpable.^27^ The stewardship approach would have involved disclosing these trade-offs upfront and listening—without defensiveness—to the backlash that followed.

Similarly, later in the pandemic, officials also admitted adjusting herd immunity estimates to encourage vaccine intake.^1^^,^^28^^,^^29^ While their intentions were arguably benevolent and protective, the use of noble lies^30^ fractured trust. Here empathetic listening would require not only admitting manipulation, but also apologizing for lying, and explaining their reasoning (and fears!) candidly, while being open to be challenged for them. Empathetic listening enables trust to be restored and grow not just from what is said, but from how officials remain present when their authority is questioned.

The shifting narratives around masks and herd immunity not only puzzled the public, but also deeply undermined the moral authority of public health institutions. To rebuild their trustworthiness,^31^ officials must now embrace a fuller communicative accountability: one where the steward (i) discloses the uncertainties and trade-offs inherent in public health decision-making with honesty and discretion; (ii) remains present when trust wavers, standing open to the relational consequences of her speech; and (iii) invites the public into the discernment process. Only then can communication answers the question, *What does this mean for you and your community?*—not as prescription, but as co-deliberation. This horizontal dynamic—where both official and public co-create understanding—embodies stewardship as a gift of presence and cultivates enduring trust.

## Conclusion

In this paper, I argued that public health officials—as stewards of the population’s well-being—have an ethical duty to convey scientific knowledge truthfully—that is, with honesty tempered by discretion. The proposed stewardship model reframes communication as a moral practice that must be accessible enough to be grasped, assessable enough to be evaluated, and accountable enough to be co-owned.

By practicing attentive presence, reasonable vulnerability, and empathetic listening, public health officials move beyond one-way directives to build relationships grounded in trust. When embedded into everyday practice, these stewardship ingredients transform communication from a tool of persuasion into a gesture of mutual learning and care.

To make this shift tangible, targeted ethics and communication training is key. Officials would benefit from sustained formation in the virtues that underpin stewardship: the discipline of loving attention, the courage to disclose limitations, and the humility to listen. Workshops and scenario-based exercises can help public health leaders learn to filter complexity, disclose uncertainty, and respond to criticism not defensively but dialogically. Restoring public trust will not come through more data or louder messaging,^16^ but through the quiet authority to those willing to speak truthfully, listen deeply, and lead with love.

## Notes

**Thana C. de Campos-Rudinsky,** Associate Professor

## References

[1] Brown RCH, de Barra. A taxonomy of non-honesty in public health communication. Public Health Ethics 2023;16:86–101. 10.1093/phe/phad003 37151785 PMC10161520

[2] Oliver I, Lewis D. Public trust is necessary to protect the population from threats to public health. J Public Health 2009;31:468–9. 10.1093/pubmed/fdp097 19897508

[3] Cordero DA . *Trust begets trust*: a reaction to a counterreaction on the issue of trust between the government and public during the COVID-19 pandemic. J Public Health 2021;43:e517–8. 10.1093/pubmed/fdab155 PMC8194595 33993278

[4] Raymond John D, Vergara PJ, Sarmiento D et al. Lagman building public trust: a response to COVID-19 vaccine hesitancy predicament. J Public Health 2021;43:e291–2. 10.1093/pubmed/fdaa282 PMC7928772 33454769

[5] WHO . *World Health Report 2000*. Geneva: WHO, 2000, viii.

[6] Brinkerhoff DW, Cross HE, Sharma S et al. Stewardship and health systems strengthening: an overview. Public Adm Dev 2019;39:4–10. 10.1002/pad.1846

[7] Saltman RB, Ferroussier Davis O. The concept of stewardship in health policy. Bull World Health Organ 2000;78:732–9. 10916910 PMC2560782

[8] Kass HD . Stewardship as a fundamental element in images of public administration. Dialogue 1988;10:2–48.

[9] Nuffield Council on Bioethics . *Public Health: Ethical Issues*. Cambridge: Cambridge Publishers, 2007, 17–8.

[10] Baldwin T, Brownsword R, Schimdt H. Stewardship, paternalism and public health: Further thoughts. Public Health Ethics 2009;2:113-6–114. 10.1093/phe/php007

[11] Coggon J . What helps us a steward? Stewardship, political theory, and public health law and ethics. Northern Ireland Legal Quart 2011;62:599–616. 10.53386/nilq.v62i5.467 PMC3605915 23526839

[12] Dawson A, Verweij M. The Steward of the Millian State. Public Health Ethics 2008;1:193–5. 10.1093/phe/phn034

[13] Coggon J . The Nanny state debate: a place where words don't do justice. Faculty Public Health 2018:7–32. https://www.fph.org.uk/policy-campaigns/publications/reports/

[14] Wilson J . *Public Health and Public Policy*, Vol. 6. Oxford: Oxford University Press, 2021.

[15] Coggon J, Tahzib F. The science of social justice: assuring the conditions for ethics and equity at the heart of public health. J Public Health 2021;43:e629–31. 10.1093/pubmed/fdaa021 32149353

[16] A Question of Trust. Cambridge: Cambridge University Press, 2002. 10.1002/cfg.220

[17] Pruss A . Lying and speaking your interlocutor’s language. Thomist 1999;63:439–53. 10.1353/tho.1999.0016

[18] Grasswick H . Understanding epistemic trust, injustice, and their harm. R Inst Philos Suppl 2018;84:78.

[19] Keohane R, Lane M, Oppenheimer M. The ethics of scientific communication under uncertainty. Politics Philos Econ 2014;13:343–68. 10.1177/1470594X14538570

[20] Massimi M . Public trust in model-based science: moving beyond the ‘view from nowhere’. In: *OUP Blog*. Oxford: Oxford Blog, 2022, 29 July. https://blog.oup.com/2022/07/public-trust-in-model-based-science-moving-beyond-the-view-from-nowhere/

[21] O’Neill OO . Ethics of communication? Eur J Philos 2009;17:167–80. 10.1111/j.1468-0378.2009.00346.x

[22] On the duty of candour, see for example: Quick O. Duties of candour in healthcare: the truth, the whole truth, and nothing but the truth? Med Law Rev 2022;30:324–47. 10.1093/medlaw/fwac004 35312762 PMC9155592

[23] WHO . Infant and Young Child Feeding. Geneva: WHO, 2021. https://www.who.int/news-room/fact-sheets/detail/infant-and-young-child-feeding

[24] Anderson E . Democracy, public policy, and lay assessments of scientific testimony. Episteme 2011;8:144–64. 10.3366/epi.2011.0013

[25] Evans CS, Rickabaugh B. Living accountability: accountability as a virtue. Int Philos Quart 2022;62:45–64. 10.5840/ipq2022519189

[26] On the central role that listening has in the practice of empathy, see Hayakawa S, Miyahara K. Empathy through listening. J Am Philos Assoc 2024.

[27] Tufekci Z . Why Telling People They Don’t Need Masks Backfired, Vol. 17. The New York Times, 2020, 2020 Published March. Retrieved 25 March 2024. https://www.nytimes.com/2020/03/17/opinion/coronavirus-face-masks.html

[28] McNeil DG Jr . *How Much Herd Immunity Is Enough*. The New York Times, 2020, 24 December 2020. Retrieved 25 March 2024. https://www.nytimes.com/2020/12/24/world/how-much-herd-immunity-is-enough.html

[29] Prasad V . *Op-Ed: Why Did Fauci Move the Herd Immunity Goal Posts?* Vol. 29. MedPage Today, 2020. https://www.medpagetoday.com/opinion/vinay-prasad/90445.

[30] *On the Concept of Noble Lies, See: Bok, Sissela. Lying: Moral Choice in Public and Private Life* . Hassocks: The Harvester Press, 1978.

[31] O’Neill OO . Linking trust to trustworthiness. Int J Philos Stud 2018;26:293–300. 10.1080/09672559.2018.1454637

